# Fusobacterium abscessus sp. nov., associated with brain abscess in humans

**DOI:** 10.1099/ijsem.0.007199

**Published:** 2026-06-12

**Authors:** Øyvind Kommedal, Diego Forni, Torbjørn Sæle Bruvold, Grete Dimmen, Marit Gjerde Tellevik, Siri Tandberg Knoop, Manuela Sironi, Audun Sivertsen

**Affiliations:** 1 Department of Microbiology, Haukeland University Hospital, Bergen, Norway; 2 Scientific Institute IRCCS E. MEDEA, Bioinformatics, Bosisio Parini, Italy; 3 School of Medicine and Surgery, University of Milano-Bicocca, Monza, Italy

**Keywords:** abscess, brain, *Fusobacterium abscessus*, *Fusobacterium nucleatum*, phylogeny, taxonomy

## Abstract

Recent genome-based phylogenetic investigations of the *Fusobacterium nucleatum* group have revealed that genomes currently annotated as *F. nucleatum* (sensu stricto) bifurcate into two separate phylogenetic groups: a large cluster containing the *F. nucleatum* type strain and a smaller cluster possibly representing a novel species. To further investigate this, we searched our strain archive for *Fusobacterium* isolates potentially representing the smaller cluster. A strain Vestland17^T^, cultured from a polymicrobial brain abscess, had been found to share less than 99% similarity with the *F. nucleatum* type strain by partial 16S rRNA gene sequencing. The isolate grew on blood agar with dry whitish colonies (0.5–1 mm) after 24–48 h of anaerobic incubation. Genome-based phylogenetic analyses confirmed that it belonged to the smaller *F. nucleatum* outlier cluster. We further confirmed the presence of this outlier cluster in four additional historic brain abscess samples using specific amplification and sequencing of the *dnaB* gene directly from remnant extracted DNA stored in our diagnostic biobank. A core-genome phylogenetic tree including the novel strain Vestland17^T^ and several more recent GenBank whole-genome references reproduced the previously observed bifurcation within the *F. nucleatum* (sensu stricto) species. Average Nucleotide Identities (ANI) between the two clusters are in the range 94.7–95.2%, supporting that the smaller cluster represents a different species as per a 95–96% ANI threshold. This was further supported by alternative strategies based on the biological species concept. For this species, we propose the name *Fusobacterium abscessus*. The type strain of *F. abscessus* is Vestland17^T^ (DSM 121298^T^, NCTC 15275^T^).

## Data Availability

Partial gene sequences amplified directly from brain abscess samples are available in GenBank with accession numbers PX664510–PX664527 (V1V2 16S rRNA gene sequences from the 2014 brain abscess study) and PX682295–PX682298 (*dnaB* from the present study).

## Introduction

Culture-independent diagnostic approaches like 16S rRNA gene-targeted next-generation sequencing have established the *Fusobacterium nucleatum* group among the most important anaerobic bacteria in human infections. They are considered key pathogens in the formation of pleural space infections and bacterial brain abscesses [1,2] and are involved in complicated appendicitis [3]. In the oral cavity, they are essential for complex dental plaque formation and among the most prevalent species in periodontal diseases and apical abscesses [4,6]. They can also be isolated from various malignant tumours, and there is considerable research focus on a potential role for these bacteria in tumour development, in particular for colorectal cancers [7,8].

*F. nucleatum* sensu stricto is the type species of the genus *Fusobacterium* [9,10]. The *F. nucleatum* group is a loosely defined clinical term encompassing the four former subspecies of *F. nucleatum* sensu lato (*F. nucleatum* subsp. *animalis*, *F. nucleatum* subsp. *nucleatum*, *F. nucleatum* subsp. *polymorphum* and *F. nucleatum* subsp. *vincentii*) and other later described closely related species [11]. It currently encompasses the seven validly published species *Fusobacterium animalis*, *Fusobacterium hwasookii*, *Fusobacterium nucleatum* (sensu stricto), *Fusobacterium paranimalis, Fusobacterium polymorphum*, *Fusobacterium vincentii* and *Fusobacterium watanabei* [12,14]. Phylogenetically, the non-human species *Fusobacterium canifelinum* and *Fusobacterium simiae* also belong to this group [12].

In clinical microbiology, the *F. nucleatum* group has been a useful term since phenotypic tests for reliable assignment to these species have not been defined [15,16] and since discrimination between several of them is also not possible based on the 16S rRNA V3–V4 region commonly used for Illumina amplicon sequencing. However, there is a growing interest in *Fusobacterium* taxonomy and niche adaptation among *Fusobacterium* species, and the lack of diagnostic resolution might have obscured species-specific disease associations [6,8, 17, 18]. *F. animalis* has been established as the dominant *Fusobacterium* species in colorectal tumours and *F. polymorphum* is associated with oral dysplasia [6,8]. More recently, it was described that, whereas *F. polymorphum* is the most abundant fusobacterial species in dental plaques, it is *F. animalis* that dominates in apical dental abscesses [4].

There are still unresolved taxonomic matters within the *F. nucleatum* group. Two recent genome-based studies have noted the existence of two genetically distinct populations among isolates currently annotated as *F. nucleatum* (sensu stricto), one large clade including the *F. nucleatum* type strain (*F. nucleatum* C1) and a smaller clade (*F. nucleatum* C2) represented by only four genomes in GenBank [8,12]. Connolly and Kelly were the first to make this observation [8]. Based on extensive phylogenetic analyses, they concluded that the smaller clade represented a new species. By reassessment of data from previous metagenomic studies, they further found this putative new species to be the third most common *Fusobacterium* species in both gingival plaques (after *F. polymorphum* and *F. vincentii*) and in stool from patients with colorectal cancer (after *F. animalis* and *F. polymorphum*) and being much more common than *F. nucleatum* C1 in both sample types. They also identified several protein families present in *F. nucleatum* C2 that were absent in *F. nucleatum* C1 and vice versa. However, this was an *in silico* study without access to bacterial strains, and no formal description was made. An eventual role in bacterial infections was also not investigated.

**Fig. 1.**
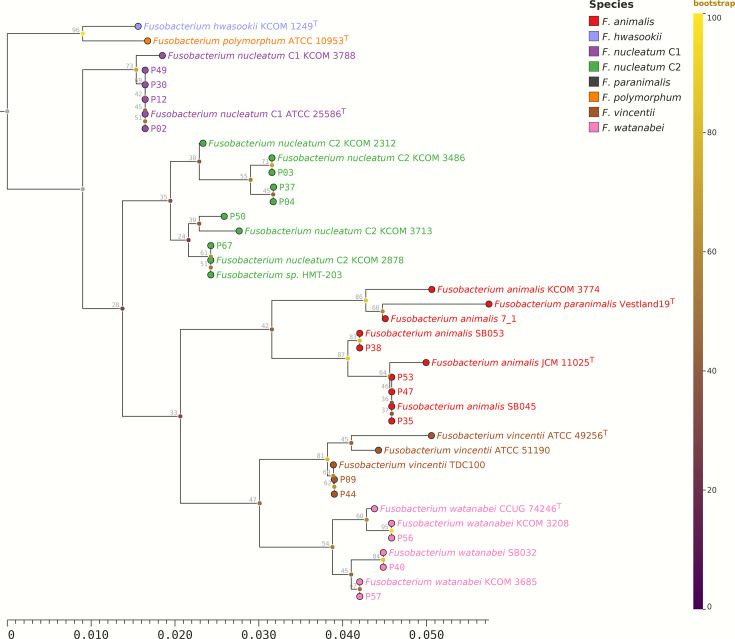
Phylogenetic tree based on the 16S rRNA V1–V2 region. The tree contains representative sequence variants for the *F. nucleatum* group species and the *Fusobacterium* sequences from the historic brain abscess study (marked with PNN).

Inspired by this, we reassessed data from a previous study where we used 16S V1–V2 targeted next-generation sequencing for the microbial characterization of bacterial brain abscesses [1]. We remembered that sequences representing the *F. nucleatum* group from these abscesses sometimes obtained the best score against the uncultured species ‘*Fusobacterium* sp. HMT-203’ from the Human Oral Microbiome Project. Indeed, upon re-analysis, we found that in 5 out of 18 abscesses containing a *F. nucleatum* group species in that study, the 16S sequence analysis resulted in the best match (99.6–100%) with the undescribed species ‘*Fusobacterium* sp. HMT-203’ and the more recently published 16S rRNA genes of the 4 genomes in the small outlier *F. nucleatum* C2 clade. In contrast, 16S rRNA gene references from the *F. nucleatum* C1 main clade shared only 97.7–98.6% identities (Fig. 1).

Unfortunately, no *Fusobacterium* strain was cultured from any of these five samples. We, therefore, reassessed findings in later brain abscesses from our diagnostic routine. In our laboratory, all brain abscess samples are routinely investigated using culture and a broad-range 16S rRNA V1–V3 amplification and Sanger sequencing approach that includes a separate 16S rRNA V1–V3 PCR for anaerobe bacteria [19]. Using this approach, from 2015 to 2025, we identified 32 brain abscesses with a *Fusobacterium* species, of which only 3 were successfully cultured. Among these, one isolate from 2017 had obtained the highest 16S rRNA gene identity with ‘*Fusobacterium* sp. HMT-203’ combined with a category B consistency for *F. nucleatum* (score 1.8) by Matrix-Assisted Laser Desorption/Ionization Time-of-Flight Mass Spectrometry (MALDI-TOF MS).

From a polymicrobial brain abscess also containing *Schaalia meyeri*, *Parvimonas micra* and *Campylobacter gracilis*, we identified a *Fusobacterium* isolate ‘Vestland 17’ that could not be validly assigned to a species in our routine diagnostics. Based on the low 16S rRNA identity with validly published *Fusobacterium* species and the low score against *F. nucleatum* protein spectra in the Bruker MALDI-TOF MS database, we suspected that this might be a representative of the recently described outlier *F. nucleatum* C2 clade, possibly representing a new species. To determine this, we set out to perform a thorough characterization of the isolate. We also sought to provide additional evidence for an association between this putative new species and brain abscess in humans.

## Methods

### Cultivation and sequencing of *Fusobacterium* sp. strain Vestland17^T^

The strain Vestland17^T^ had been stored in −80 °C since it was first cultured from a brain abscess in 2017. After thawing, it was cultivated on Columbia blood agar plates (Liofilchem, Roseto degli Abruzzi, Italy) for 48 h in a strict anaerobe atmosphere at 35 °C. Genomic DNA extraction was performed from bacterial colonies using the TANBead Maelstrom 4800 automated extractor (Taiwan Advanced Nanotech, Taoyuan, Taiwan). The ONT library preparation was performed using Ligation Sequencing Kit V14 (SQK-LSK114, Oxford Nanopore, Oxford, UK), and sequencing was performed using a R10.4.1 Flongle Flow cell. The genome was subsequently *de novo* assembled using Hybracter [20] with 67× coverage and annotated using Bakta v. 1.9.2 [21].

### Generation of a MALDI-TOF MS reference spectrum

A MALDI-TOF MS reference spectrum for *Fusobacterium abscessus* strain Vestland17^T^ was generated using fresh colonies (48-h incubation) according to instructions provided by Bruker.

### Amplification and sequencing of partial *dnaB* from clinical samples

For four of the remaining 29 brain abscess samples from the 2015–2025 period, from which a *Fusobacterium* species had been detected by 16S rRNA broad-range amplification and sequencing, but no strain was cultured, the 16S rRNA sequence analysis indicated the potential new species *F. nucleatum* C2.

To confirm this, we designed a real-time PCR targeting a 494 bp segment of the fusobacterial *dnaB* gene, providing better discrimination within the *F. nucleatum* group. Several other alternative gene targets were evaluated, including the previously used *znpA* [4] and *rpoB* [22], but *dnaB* provided the most consistent discrimination while at the same time allowing for the design of a sensitive and specific PCR. The *dnaB* target was amplified from remnant DNA eluate stored in our diagnostic biobank at −80 °C. The 25 µl real-time PCR reaction mixture consisted of 12.5 µl of TB Green Premix Ex Taq mastermix (TaKaRa Bio, Otsu, Japan), 1 µl forward primer (Fuso_dnaB-F: AATGATGTCAGAGTRGCAGAAGA), 1 µl reverse primer (Fuso_dnaB-R: GTATGAGGTAAATCGGCAACAA), 2 µl template and 8.5 µl PCR-grade water. Amplification was carried out on a QuantStudio5 (QIAGEN) real-time PCR apparatus using the following thermal profile: an initial enzyme activation step (30 s/95 °C) followed by 45 cycles of DNA denaturation (10 s/95 °C), primer-annealing (10 s/60 °C) and elongation (20 s/72 °C). Sanger sequencing of the PCR product was performed using an ABI Prism 1.1 Big Dye sequencing kit and an ABI 3730 DNA analyser (Applied Biosystems, Foster City, California).

### Accession numbers and phylogenetic analyses

For the genome-based phylogenetic analyses, we included all *F. nucleatum* group whole genomes used in our recent assessment of the *F. nucleatum* group phylogeny [12]. In addition, we expanded the *F. nucleatum* C1/C2 clusters with 18 new genomes, including the genomes for *Fusobacterium* sp. strain Vestland17^T^ and *Fusobacterium* sp. HMT-203 strain W7671. All included genomes and partial gene segments used in phylogenetic comparisons are listed in Table S1 (available in the online Supplementary Material) together with their respective accession numbers.

### Core gene analysis

The Genome Database Taxonomy toolkit (GTDB-Tk, v.2.5.2) was used to identify and extract protein sequences for a set of 120 single-copy marker genes that are known to be conserved among bacteria [23]. GTDB-Tk also generated a multiple sequence alignment based on this marker set by retaining a maximum number of columns per gene (50 in our case). This variant alignment was then used as input to build a phylogenetic tree with IQ-TREE [24]. The best substitution model (JTT+F+R7) was automatically selected by the ModelFinder tool implemented in IQ-TREE v.1.6.12 [25]. Robustness of the final tree was assessed using 1,000 bootstrap replicates [26]. All other parameters were set to the default.

### Average nucleotide identity and the biological species concept methods

Skani v.0.3.1 [27] was used to calculate all-versus-all matrices of all genomes, as well as to show Average Nucleotide Identity (ANI) species discontinuity through comparisons to reference strains using the ‘skani dist’ command. All ANI plots were made using R with ggplot2 [28] and ComplexHeatmap [29]. *Fusobacterium* populations were also characterized using PopCOGenT [30], a network-based tool designed to detect horizontal gene transfer (HGT). Specifically, PopCOGenT defines species delimitations by identifying recent HGT events through the detection of regions of high-sequence identity between genomes. PopCOGenT calculates a parameter known as ‘length bias’, which quantifies how the observed distribution of identical genomic regions between two genomes deviates from an expected null model without recombination [30]. Thus, PopCOGenT infers population structure and constructs gene-flow networks, where each strain (or clonal cluster) is represented as a node, and the edge thickness is proportional to the amount of gene flow. We ran PopCOGenT using all the *Fusobacterium* strains in our dataset, and we plotted gene flow networks using Cytoscape v3.9.1 [31].

Biological species identification was also carried out using the ConSpeciFix tool [32]. This tool is based on the analysis of core genes shared by a defined group of genomes and evaluates the ratio of homoplastic/recombinant polymorphisms (h) to mutations (m) across them. To determine the species assignment of a single genome, its sequence is compared to a set of genomes that are already classified as a single species, and the h/m ratio is calculated. If the tested strain belongs to a different species, h/m ratios will decrease [32].

To investigate the relationship between *F. nucleatum* C1 and *F. nucleatum* C2, we ran ConSpeciFix using the ‘personal comparison’ method, by running *F. nucleatum* C1 strains alone and *F. nucleatum* C1 strains plus the Vestland17^T^ strain.

### Partial gene segment comparisons

The partial 16S rRNA (V1V2) gene and *dnaB* sequences obtained by direct amplification and Sanger sequencing from clinical brain abscess samples were aligned with corresponding representative partial sequences from members of the *F. nucleatum* group downloaded from GenBank. Genetic distances were calculated using the Tamura–Nei model and evaluated by bootstrap analysis with 500 replications. The neighbour-joining method was used for reconstruction of phylogenetic trees.

## Results and discussion

**Fig. 2.**
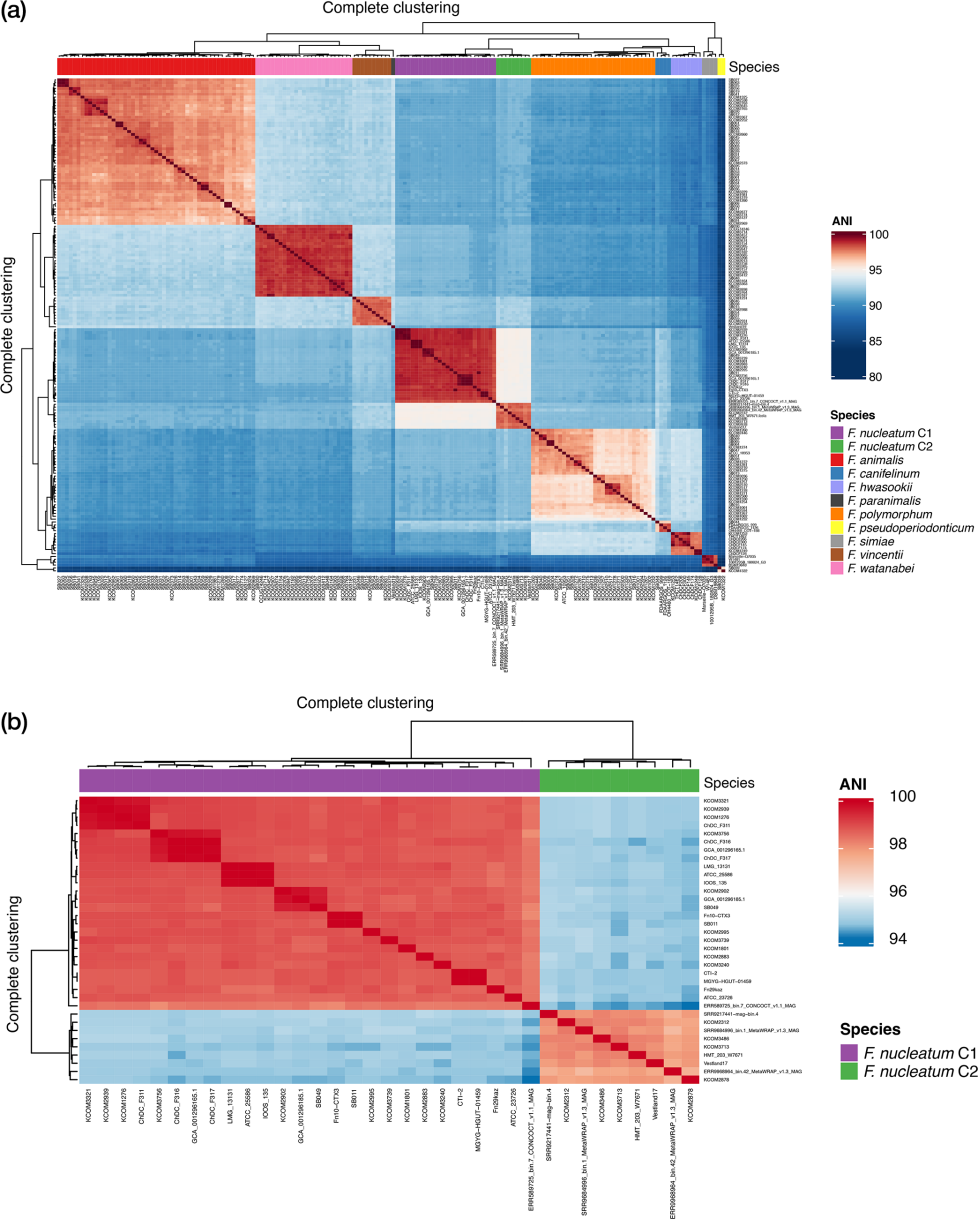
ANI comparisons between *Fusobacterium* spp. (**a**) Heatmap of ANI differences between genomes within the *F. nucleatum* group, as determined by SKANI, with colour shading highlighting the canonical 95% ANI species delineation rule. (**b**) Heatmap of ANI differences for *F. nucleatum* and *F. abscessus*, showing that they fall below a 96% threshold.

**Fig. 3.**
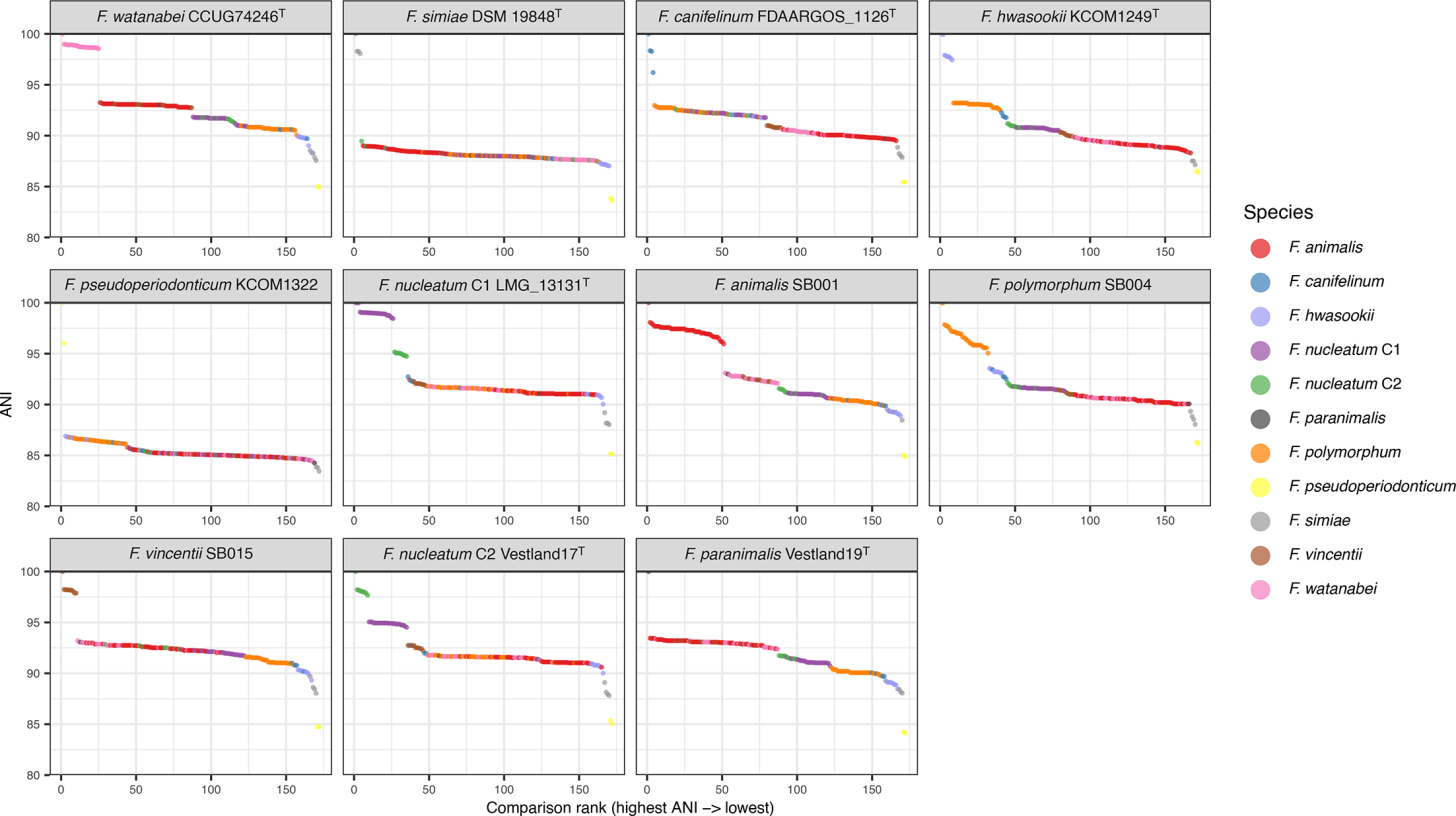
Demonstration of ANI discontinuity between species of the *F. nucleatum* group. Each plot shows the ANI of a representative strain of each species (either reference strains or genomes with closed circular chromosomes) compared to all other strains of the dataset, starting with a self-self comparison. Strains are coloured according to species as shown in the legend.

All genomes were included in an all-vs-all ANI calculation using SKANI [33]. A heatmap (Fig. 2a) shows that *Fusobacterium* sp. strain Vestland17^T^, *Fusobacterium* sp. HMT-203 strain W7671 and three more of the extra genomes included in this study cluster together with the previously noted *F. nucleatum* C2 genomes, forming a cluster of nine genomes that is separated from the *F. nucleatum* C1 main cluster containing the *F. nucleatum* type strain. ANI identities between the two clusters are in the range 94.7–95.2% (Fig. 2b), supporting that they are different species as per a 95–96% ANI threshold [34]. While this is a smaller distance than what has previously been reported between species within the *Fusobacterium nucleatum* group, it is not fundamentally different. Previously, the two most closely related species were *F. animalis* and *F. paranimalis* with interspecies ANI identities in the range 93.0–93.5%. With only a single genome available for *F. paranimalis*, the gap between these two species is likely to be further reduced with the future inclusion of more *F. paranimalis* strains. The ANI discontinuity plots (Fig. 3), motivated by the work of Passarelli-Araujo *et al*. [35], show a clear drop in pair-wise ANI values when the closest genome from *F. nucleatum* C1 is included in the *F. nucleatum* C2 cluster and vice versa, and further that this drop is similar in size to the drop observed for *F. animalis* and larger than the drop for *F. polymorphum* with the inclusion of a genome from their nearest neighbouring species. Attempts to quantize discontinuity ranges between species with statistical support, e.g. with Jack-knifing approaches, were not done as there are not sufficient genomes within all species to motivate such calculations [35].

**Fig. 4.**
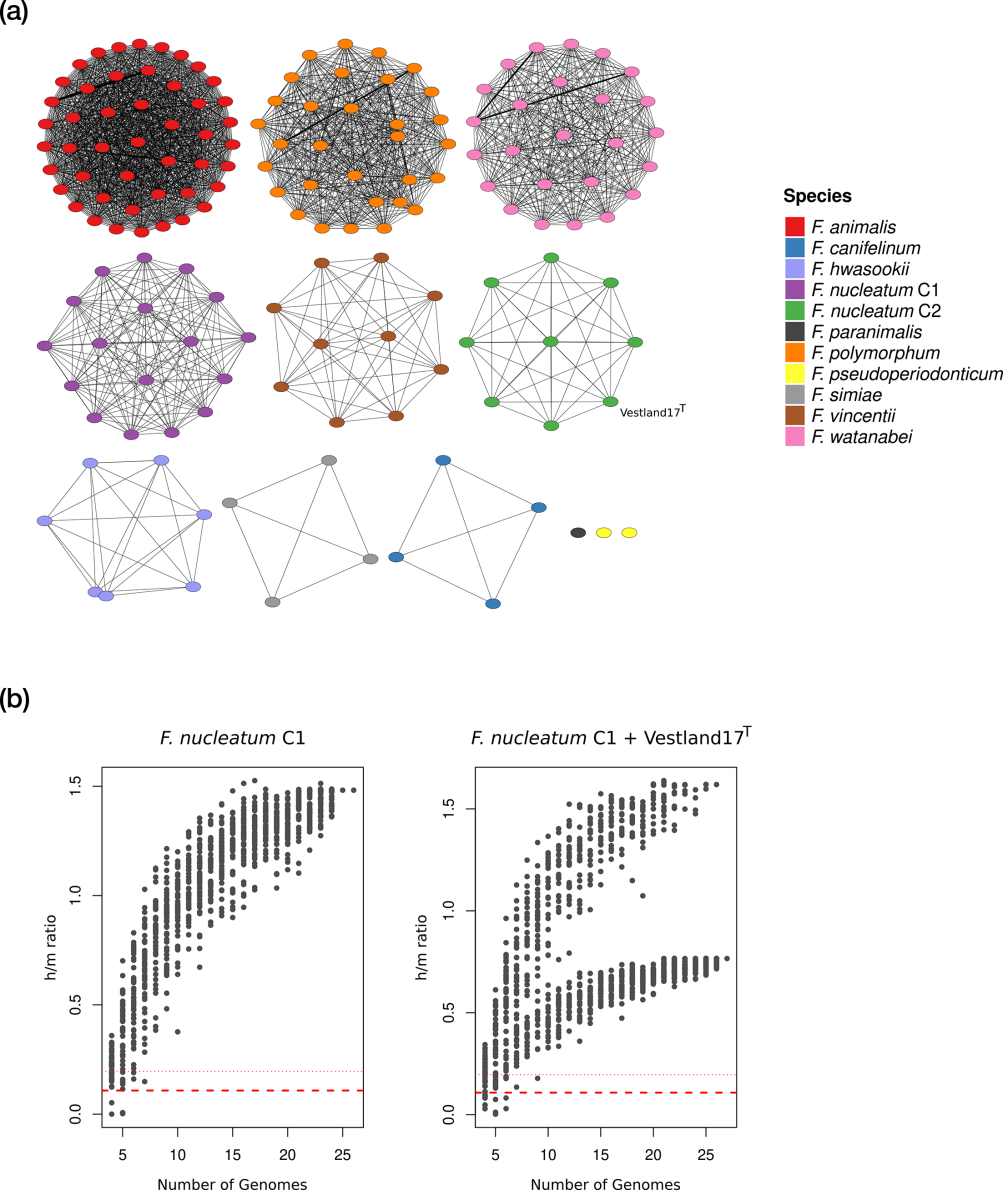
Gene flow between *Fusobacterium* genomes. (**a**) PopCOGenT analysis. Nodes represent bacterial strains, and edges represent the inferred amount of gene flow between them (expressed in terms of length bias). Edge width is proportional to the amount of gene flow between pairs of genomes. Clonal clusters were collapsed into single nodes. (**b**) Homologous recombination between *Fusobacterium* species, as determined with ConSpeciFix. Homoplasy/mutation ratios of *F. nucleatum* or *F. nucleatum* plus Vestland17^T^ are plotted. A single curve indicates the presence of one species, whereas the presence of a second lower curve indicates that two different species are present in the sample. Red lines indicate the expected average and maximal h/m ratios when all homoplasies are introduced only by convergent mutations.

To further support the separation of *F. nucleatum* C2 as a new species different from *F. nucleatum* C1, we applied an alternative strategy based on the biological species concept, as described previously [12]. The biological species concept defines a species as a group of interbreeding individuals that remain reproductively isolated from other groups. For bacteria, gene-flow discontinuities are used to define biological species. The whole data set of *Fusobacterium* genomes was used as an input for ‘populations as clusters of gene transfer’ (PopCOGenT) [30]. PopCOGenT can detect recent gene-flow discontinuities and use this to delineate species. As previously described, PopCOGenT identified 12 genetically isolated ecological units with no detectable gene-flow among them (Fig. 4a) and defined clusters with excellent congruence to ANI-defined species. PopCOGenT results thus support that the nine *F. nucleatum* C2 genomes, which include Vestland17^T^, represent a separate biological species from *F. nucleatum* C1.

These results were also confirmed by using an approach that relies on the detection of homoplasies [32]. Based on the h/m parameter, *F. nucleatum* C1 strains showed high levels of homoplastic polymorphisms across core genomes, implying a single biological species (Fig. 4b). However, the inclusion of the Vestland17^T^ strain resulted in a sharp drop of h/m values, a signature of the presence of two distinct species (Fig. 4b).

**Fig. 5.**
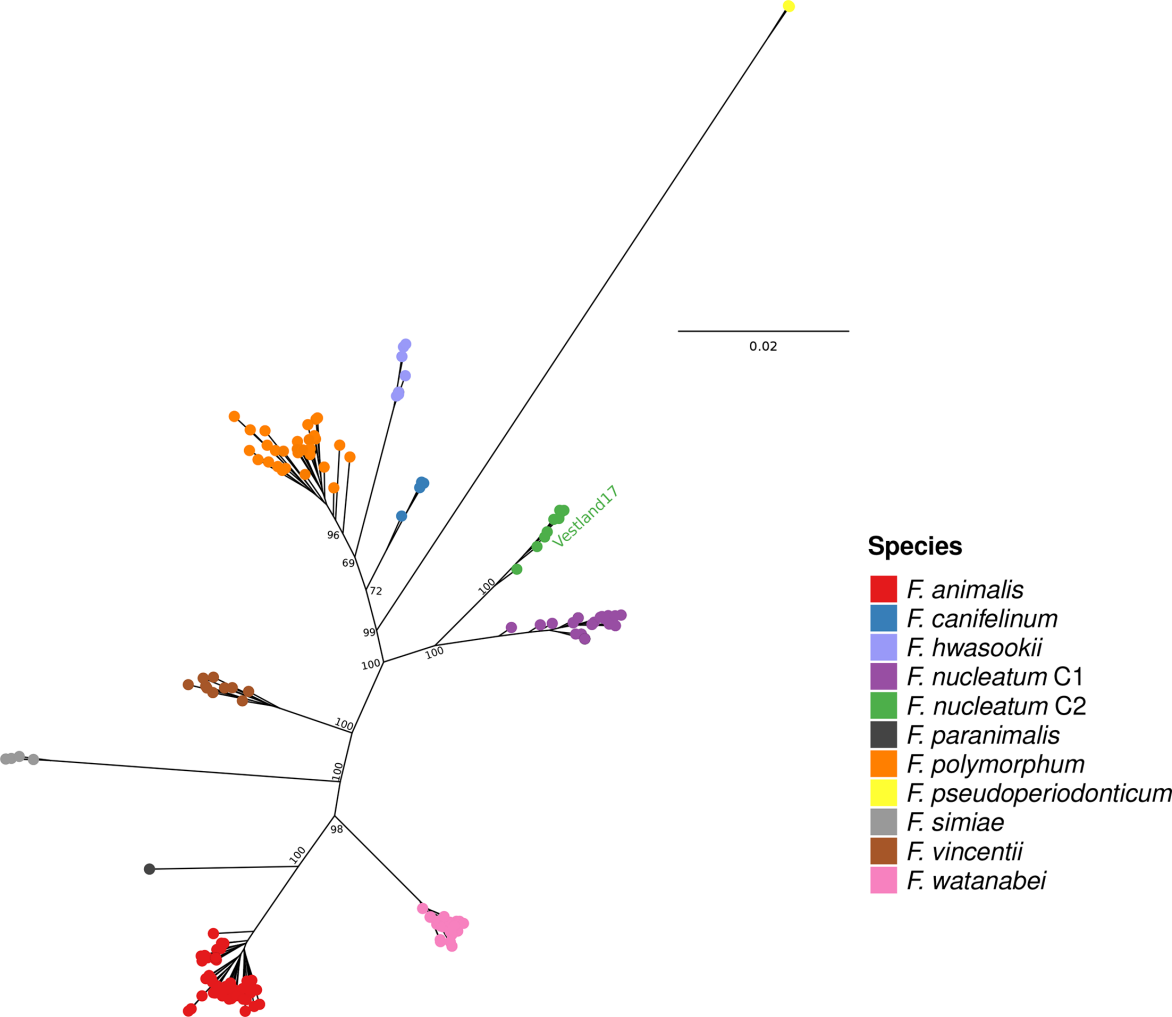
Phylogenetic relationship among *Fusobacterium* species. Maximum-likelihood tree based on 120 conserved proteins. Strains are coloured according to the legend and the *Fusobacterium* strain isolated in this work is highlighted. Bootstrap percentage is also reported for relevant nodes.

Finally, we used the GTDB-Tk to extract the sequences of 120 core genes present in the *F. nucleatum* group genomes. The alignment of the core genes was used to generate a phylogenetic tree, recapitulating the phylotaxonomic order determined by the analyses above, including the split of *F. nucleatum* into two lineages (Fig. 5).

Based on the above analyses, we conclude that *F. nucleatum* C2 is a separate species from *F. nucleatum* C1 containing the *F. nucleatum* type strain. For this novel species, we propose the name *Fusobacterium abscessus* sp. nov.

*F. abscessus* can be readily identified and discriminated from other closely related *Fusobacteria*, including *F. nucleatum,* using MALDI-TOF MS (Fig. S1). Interestingly, a reference spectrum likely representing *F. abscessus* is already present in the Bruker database, currently annotated ‘*Fusobacterium* sp. 110324_90 PNU’, providing a category A score of 2.3 when analysing the Vestland17^T^ strain. For current Bruker references with a valid species name, the best score for strain Vestland17^T^ is obtained against *F. nucleatum* subsp. *fusiforme* DSM 19508^T^, providing a category B score of 1.8. Today, *F. nucleatum* subsp. *fusiforme* is considered a later synonym of *F. nucleatum* subsp. *vincentii,* and the strain has been renamed as *F. nucleatum* subsp. *vincentii* DSM 19508 [36]. Comparisons between MALDI-TOF MS spectra for *F. abscessus* Vestland17^T^ and *Fusobacterium vincentii* DSM 19508, as well as the type strains of *F. nucleatum*, *F. watanabei* and *F. paranimalis,* are provided in Fig. S1.

Representatives of both *F. nucleatum* and *F. abscessus* carry five 16S rRNA gene copies. However, based on available genomes, there is a notable difference in their 16S rRNA gene population structures. While intra- and inter-strain 16S rRNA gene variability within *F. nucleatum* is only 0–5 nt, corresponding to 99.7–100% identities, a larger variability is present within *F. abscessus* (0–13 nt, 99.1–100% identities). Interestingly, the majority of *F. abscessus* 16S rRNA variations are localized to 10 positions in a short region from base 64 to 204 (Fig. S2). In *F. nucleatum*, this region appears stable with no variability in the 10 positions. Due to these variabilities, complete 16S rRNA gene identities between *F. nucleatum* and *F. abscessus* can be in the range 98.9–99.5%. As described in the introduction, sequence similarity in the 16S rRNA V1–V2 region is lower. Positions 89, 189 and 196 may be used to discriminate *F. nucleatum* (89C, 189G and 196C) from all *F. abscessus* variants (89A, 189 A/T and 196 A/T).

The availability of a single *F. abscessus* isolate restricted phenotypic characterization, which might be a motivation for future work.

### Clinical relevance

*Fusobacterium abscessus* is associated with bacterial brain abscess in humans. In our historic material from a Norwegian prospective study published in 2014 [1], as indicated by the 16S rRNA gene V1–V2 region, it was the most prevalent sequence variant detected (5/18), followed by *F. nucleatum* and *F. animalis* (both 4/18), *F. watanabei* (3/18) and *F. vincentii* (2/18).

Due to the relatively low number of available genomes for *F. nucleatum* and *F. abscessus*, combined with the low number of discriminating positions in the 16S rRNA gene (with corresponding low bootstrap values in Fig. 1), we used direct *dnaB* gene amplification and sequencing from four additional brain abscesses to confirm the relevance of *F. abscessus* in this life-threatening purulent cerebral infection (Fig. S3). These four samples were identified among 32 later brain abscess samples with a *F. nucleatum* group species, analysed in our diagnostic routine in the period from 2015 to 2025. They were selected since the original anaerobe 16S rRNA V1–V3 chromatogram from the diagnostic routine was a non-mixed chromatogram representing only a single bacterial sequence, clearly indicative of *F. abscessus*. In addition, comes the isolation of strain Vestland17^T^ from another sample in the same collection. However, the actual proportion of *F. abscessus* in this material was not determined and might be higher than 5/32, since most of the *F. nucleatum* group identifications were based on analysis of mixed chromatograms [37] representing multiple anaerobe bacteria that did not allow for further assignments to the species level. An eventual role of *F. abscessus* in other human infections remains to be established. In diagnostic microbiology, *F. abscessus* can be identified using Sanger sequencing of the 16S rRNA V1–V3 region with focus on the signature base positions described above. Protein spectra comparisons with closely related type strains suggest that, provided an appropriate reference spectrum, *F. abscessus* also can be identified to the species level using MALDI-TOF MS.

## Description of *Fusobacterium abscessus* sp. nov.

*Fusobacterium abscessus* sp. nov. (ab.sces’sus. L. gen. n. *abscessus*, of an abscess) is a slender fusiform anaerobic gram-negative rod-shaped bacterium, most closely related to *F. nucleatum*. It grows with dry whitish colonies (0.5–1 mm) after 24–48 h of incubation in a strict anaerobe atmosphere at 35 °C.

*F. abscessus* is a commensal species from the human oral microbiota. By metagenomic analysis, it has been found in sub- and supra-gingival plaque, but not in saliva samples [9]. It includes the strain ‘*Fusobacterium* sp. HMT-203 strain W7671’ from the Human Oral Microbiome Project (https://homd.org/). Like several other species within the *F. nucleatum* group, it is a pathobiont that can be involved in human purulent infections, and it appears to be common in brain abscesses seeded from a focus in the oral cavity. By metagenomic analysis, it has also been demonstrated to be elevated in stool samples from patients with colorectal cancer and Crohn’s disease.

The type strain is *F. abscessus* Vestland17^T^ (NCTC 15275^T^ and DSM 121298^T^). The strain was isolated from a polymicrobial brain abscess at Haukeland University Hospital, Bergen, Norway, in 2017. The chromosome size is 2,209,468 bp and includes 48 tRNAs, 15 rRNAs and 2,052 CDSs when annotated with Bakta. The G+C content is 27 mol%. The GenBank accession number for the full-length 16S rRNA gene sequence is PX636095. This whole genome has been deposited at DDBJ/ENA/GenBank under the accession JBSPTX000000000. The version described in this paper is version JBSPTX010000000.

## Supplementary material

10.1099/ijsem.0.007199 Supplementary Material 1.

10.1099/ijsem.0.007199 Supplementary Material 2.

## Abbreviations

GTDB-Tk: Genome Taxonomy Database Toolkit
HGT: horizontal gene transfer

## References

[1] Kommedal Ø, Wilhelmsen MT, Skrede S, Meisal R, Jakovljev A (2014). Massive parallel sequencing provides new perspectives on bacterial brain abscesses. J Clin Microbiol.

[2] Dyrhovden R, Eagan TM, Fløtten Ø, Siljan W, Leegaard TM (2023). Pleural empyema caused by *Streptococcus intermedius* and *Fusobacterium nucleatum*: a distinct entity of pleural infections. *Clin Infect Dis*.

[3] Vanhatalo S, Munukka E, Kallonen T, Sippola S, Grönroos J (2022). Appendiceal microbiome in uncomplicated and complicated acute appendicitis: a prospective cohort study. PLoS One.

[4] Krieger M, AbdelRahman YM, Choi D, Palmer EA, Yoo A (2024). Stratification of *Fusobacterium nucleatum* by local health status in the oral cavity defines its subspecies disease association. Cell Host Microbe.

[5] Kolenbrander PE, London J (1993). Adhere today, here tomorrow: oral bacterial adherence. J Bacteriol.

[6] Wolf M, Steinberg T, Scholz KJ, Kruse A, Rezasoltani S (2025). The rise and evolving role of *Fusobacterium nucleatum* subspecies. *Curr Res Microb Sci*.

[7] Forni D, Sivertsen A, Cagliani R, Mozzi A, Molteni C (2025). Positive selection at core genes may underlie niche adaptation in Fusobacterium animalis. Gut Pathog.

[8] Connolly JP, Kelly L (2025). The physical biogeography of *Fusobacterium nucleatum* in health and disease. mBio.

[9] Knorr M (1922). Über die fusospirillare symbiose, die gattung fusobacterium (k.b. lehmann) und spirillum sputigenum. Zugleich ein beitrag zur bakteriologie der mundhöhle. II. Mitteilung. die gattung *Fusobacterium*. Zentralbl Bakteriol Parasitenkd Infektionskr Hyg.

[10] Skerman VBD, McGowan V, Sneath pHA (1980). Approved lists of bacterial names. Int J Syst Evol Microbiol.

[11] Otero JA, Mandrekar J, Wolf MJ, Starkey JC, Carmona EM (2024). Pleural space infection microbiology as assessed using a clinical sequencing-based assay: *Fusobacterium nucleatum* group, *Streptococcus intermedius,* and other oral normal microbiota are the most common bacteria identified in community-acquired pleural space infections. J Clin Microbiol.

[12] Sivertsen A, Forni D, Molteni C, Bivand J, Dimmen G (2025). Reassessing taxonomy and virulence in the *Fusobacterium nucleatum* group—rebuttal of *Fusobacterium animalis* clades “fna C1” and “fna C2,” genome announcement for *Fusobacterium watanabei*, and description of *Fusobacterium paranimalis* sp. nov. mBio.

[13] Kook J-K, Park S-N, Lim YK, Cho E, Jo E (2017). Genome-based reclassification of *Fusobacterium nucleatum* subspecies at the species level. Curr Microbiol.

[14] Tomida J, Akiyama-Miyoshi T, Tanaka K, Hayashi M, Kutsuna R (2021). *Fusobacterium watanabei* sp. nov. As additional species within the genus *Fusobacerium*, isolated from human clinical specimens. Anaerobe.

[15] Kim H-S, Lee D-S, Chang Y-H, Kim MJ, Koh S (2010). Application of rpoB and zinc protease gene for use in molecular discrimination of *Fusobacterium nucleatum* subspecies. J Clin Microbiol.

[16] Gharbia SE, Shah HN (1990). Heterogeneity within *Fusobacterium nucleatum*, proposal of four subspecies. Lett Appl Microbiol.

[17] Bi D, Wu Y, Ji G, Zhu X, Li H (2026). Integrating ANI and phylogenies for re-evaluation of *Fusobacterium* taxonomy and disease associations. *Nat Commun*.

[18] Wolf M, Scholz KJ, Al-Ahmad A, Steinberg T, Kruse A (2026). Beyond a single species: mapping virulence traits across the redefined *Fusobacterium nucleatum* complex. Virulence.

[19] Kommedal Ø, Lekang K, Langeland N, Wiker HG (2011). Characterization of polybacterial clinical samples using a set of group-specific broad-range primers targeting the 16S rRNA gene followed by DNA sequencing and RipSeq analysis. *J Med Microbiol*.

[20] Bouras G, Houtak G, Wick RR, Mallawaarachchi V, Roach MJ (2024). Hybracter: enabling scalable, automated, complete and accurate bacterial genome assemblies. Microb Genom.

[21] Schwengers O, Jelonek L, Dieckmann MA, Beyvers S, Blom J (2021). Bakta: rapid and standardized annotation of bacterial genomes via alignment-free sequence identification. Microb Genom.

[22] Bi D, Zhu Y, Gao Y, Li H, Zhu X (2022). Profiling *Fusobacterium* infection at high taxonomic resolution reveals lineage-specific correlations in colorectal cancer. Nat Commun.

[23] Chaumeil P-A, Mussig AJ, Hugenholtz P, Parks DH (2022). GTDB-Tk v2: memory friendly classification with the genome taxonomy database. Bioinformatics.

[24] Minh BQ, Schmidt HA, Chernomor O, Schrempf D, Woodhams MD (2020). IQ-TREE 2: new models and efficient methods for phylogenetic inference in the genomic era. Mol Biol Evol.

[25] Nguyen L-T, Schmidt HA, von Haeseler A, Minh BQ (2015). IQ-TREE: a fast and effective stochastic algorithm for estimating maximum-likelihood phylogenies. Mol Biol Evol.

[26] Hoang DT, Chernomor O, von Haeseler A, Minh BQ, Vinh LS (2018). UFBoot2: improving the ultrafast bootstrap approximation. Mol Biol Evol.

[27] Shaw J, Yu YW (2023). Fast and robust metagenomic sequence comparison through sparse chaining with skani. Nat Methods.

[28] Wickham H (2016). Ggplot2, elegant graphics for data analysis.

[29] Gu Z (2022). Complex heatmap visualization. *iMeta*.

[30] Arevalo P, VanInsberghe D, Elsherbini J, Gore J, Polz MF (2019). A reverse ecology approach based on a biological definition of microbial populations. Cell.

[31] Shannon P, Markiel A, Ozier O, Baliga NS, Wang JT (2003). Cytoscape: a software environment for integrated models of biomolecular interaction networks. *Genome Res*.

[32] Bobay L-M, Ochman H (2017). Biological species are universal across Life’s domains. Genome Biol Evol.

[33] Shaw J, Yu YW (2023). Fast and robust metagenomic sequence comparison through sparse chaining with skani. Nat Methods.

[34] Riesco R, Trujillo ME (2024). Update on the proposed minimal standards for the use of genome data for the taxonomy of prokaryotes. Int J Syst Evol Microbiol.

[35] Passarelli-Araujo H, Venancio TM, Hanage WP (2025). Relating ecological diversity to genetic discontinuity across bacterial species. Genome Biol.

[36] Kook J-K, Park S-N, Lim YK, Choi M-H, Cho E (2013). *Fusobacterium nucleatum* subsp. fusiforme Gharbia and Shah 1992 is a later synonym of *Fusobacterium nucleatum* subsp. vincentii Dzink et al. 1990. *Curr Microbiol*.

[37] Kommedal Ø, Karlsen B, Saebø Ø (2008). Analysis of mixed sequencing chromatograms and its application in direct 16S rRNA gene sequencing of polymicrobial samples. J Clin Microbiol.

