# Supplementary material for: Fusobacterium abscessus sp. nov., associated with brain abscess in humans
Source: Int J Syst Evol Microbiol. 2026 Jun 12;76(6):007199. doi: 10.1099/ijsem.0.007199 (PMC13262755; doi:10.1099/ijsem.0.007199)
Supplement: Supplementary Material 1. [file ijsem-76-07199-s001.pdf]

Supplementary Figure 1: Protein spectra (MALDI-TOF MS) comparisons between *F. abscessus* and the most closely related *Fusobacterium* species/strains including a MALDI-TOF MS dendrogram.

A)

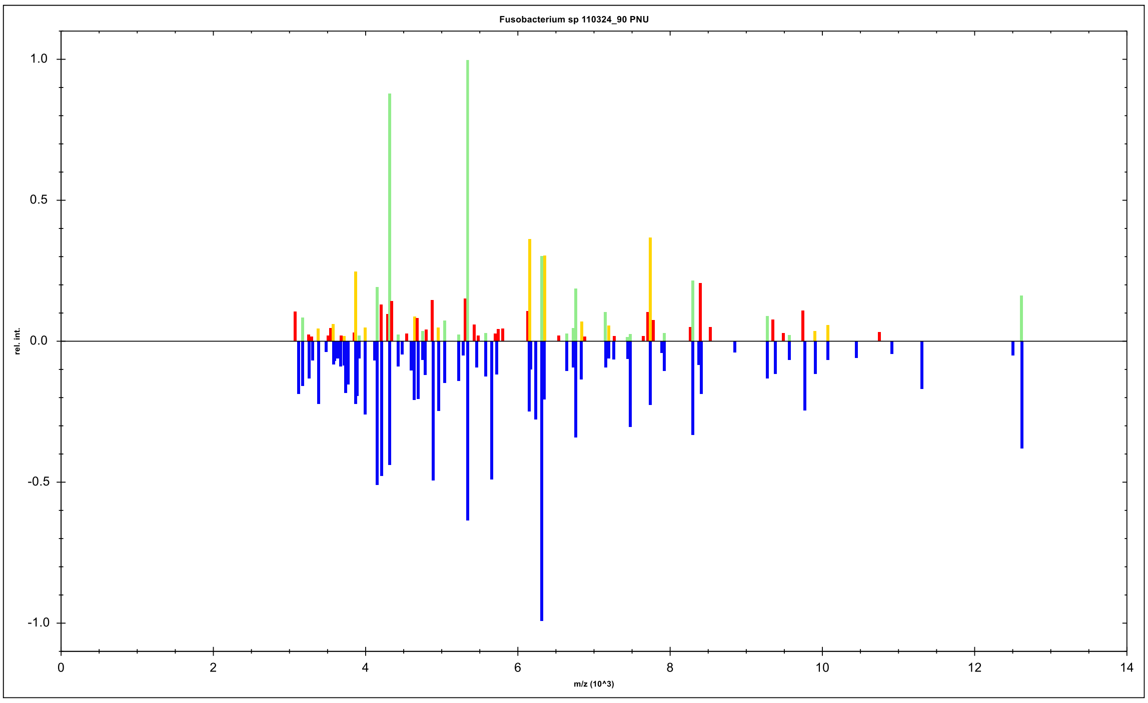

B)

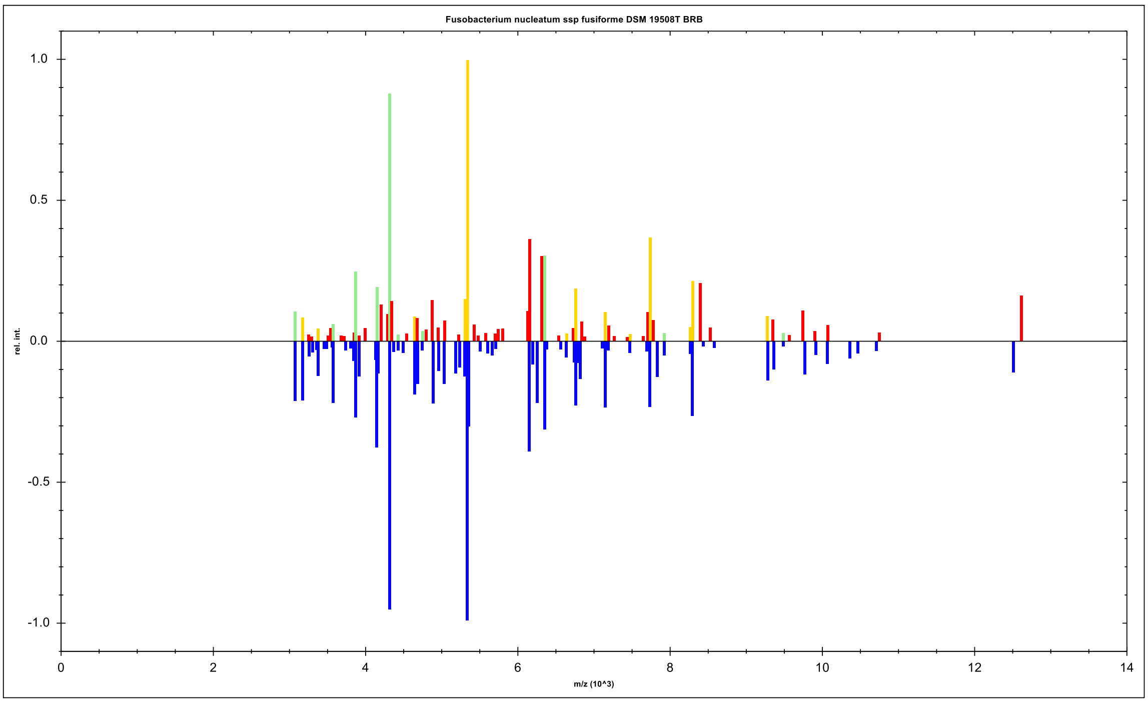

C)

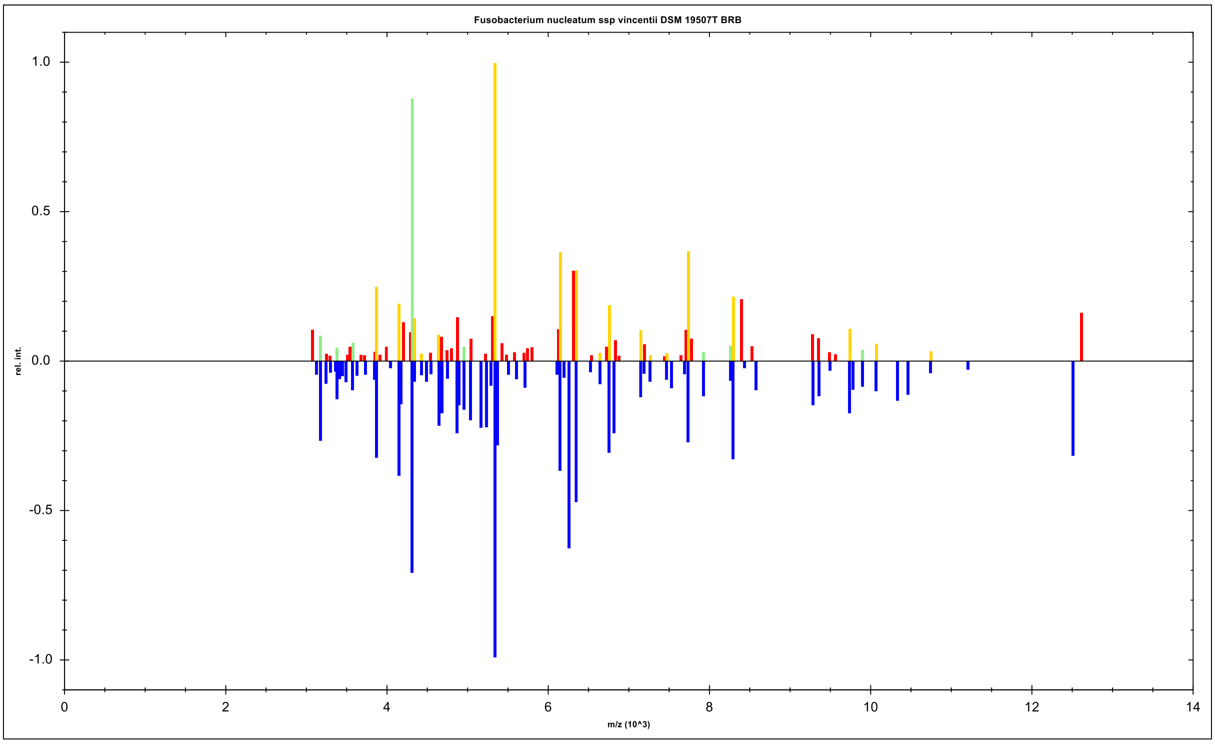

Fusobacterium nucleatum ssp vincentii DSM 19507T BRB
1.0
0.5
0.0
-0.5
-1.0
rel. int.
0
2
4
6
8
10
12
14
m/z (10^3)

D)

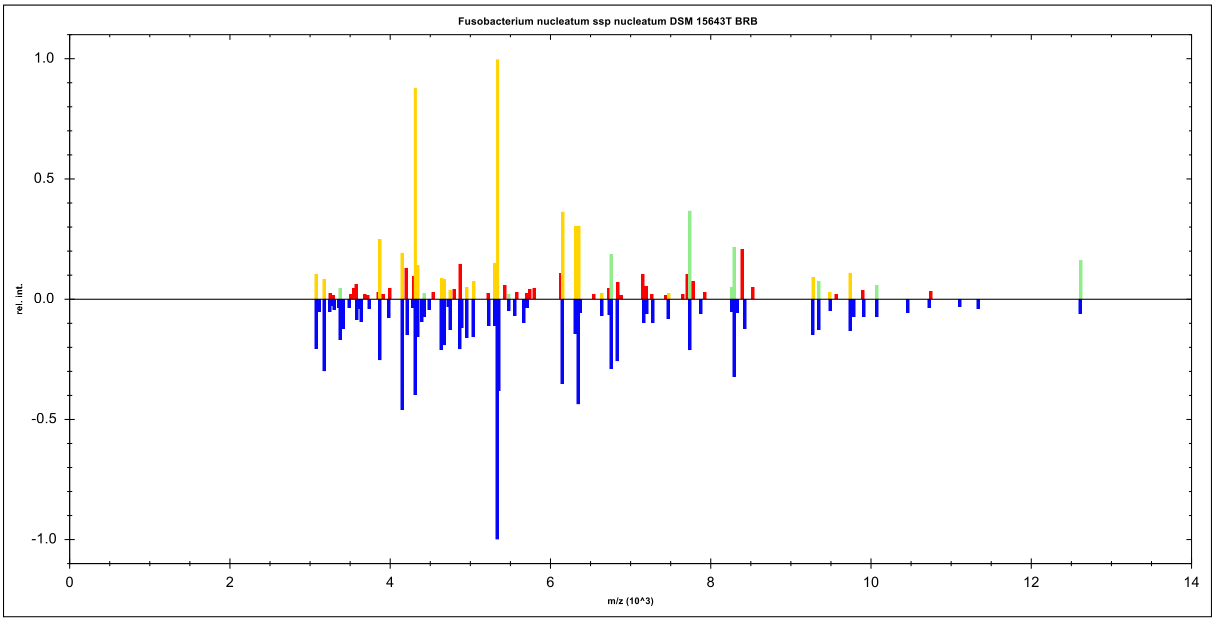

Fusobacterium nucleatum ssp nucleatum DSM 15643T BRB
1.0
0.5
0.0
-0.5
-1.0
rel. int.
0
2
4
6
8
10
12
14
m/z (10^3)

E)

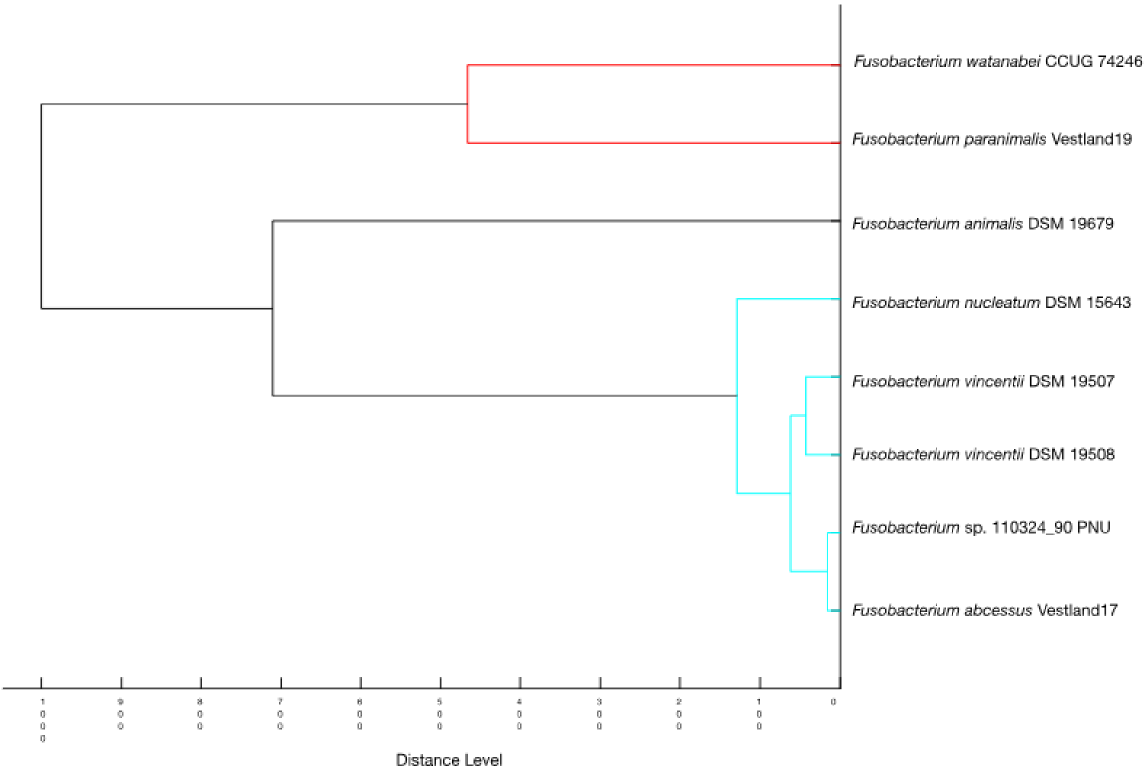

Comparisons between *F. abscessus* Vestland17^T (upper spectrum) and A) *Fusobacterium* sp. 110324_90 PNU, B) *Fusobacterium nucleatum* subsp. *fusiforme* DSM 19508^T, which has later been renamed as *F. vincentii* DSM 19508, C) *Fusobacterium vincentii* DSM 19507^T and D) *Fusobacterium nucleatum* DSM 15643^T. Green color indicates a perfect peak match, yellow indicates a poor peak match and red no peak match against the respective database reference spectra (blue peaks). E) MALDI-TOF MS dendrogram showing discrimination between *Fusobacterium abscessus* (including *Fusobacterium* sp. 110324_90 PNU) and closely related species.

Supplementary Figure 2: 16S rRNA gene variable positions in *Fusobacterium abscessus*

a) Sorted by strain after increasing intra strain variability

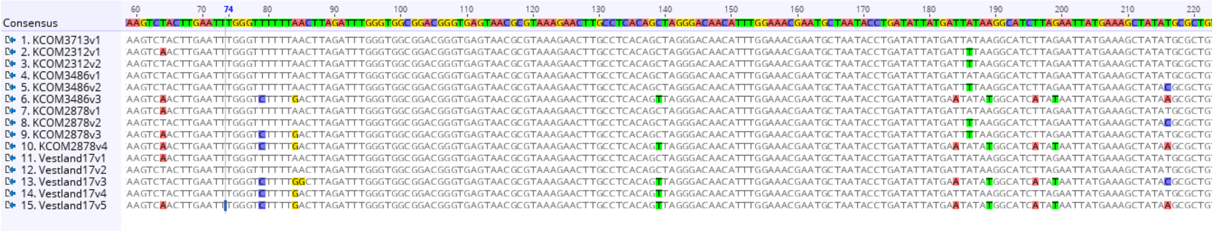

b) Sorted by mutations

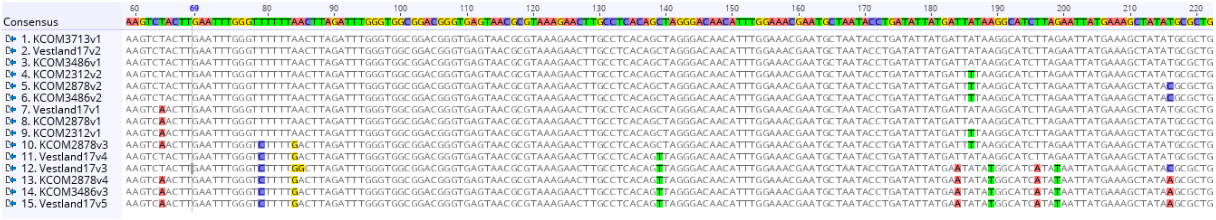

The figure includes the four *F. abscessus* sp. nov. strains from our previous study and the novel strain Vestland19^T. All 16S rRNA variants from each genome are included (indicated by v1-v5). The largest intra-strain variations were observed for strains Vestland17^T, KCOM3486 and KCOM2878. The genome for "*Fusobacterium* sp. HMT-203" was sequenced using short-read Illumina sequencing only and therefore excluded from 16S rRNA gene population comparisons.

Supplementary Figure 3: Phylogenetic tree based on the partial *dnaB* gene segment

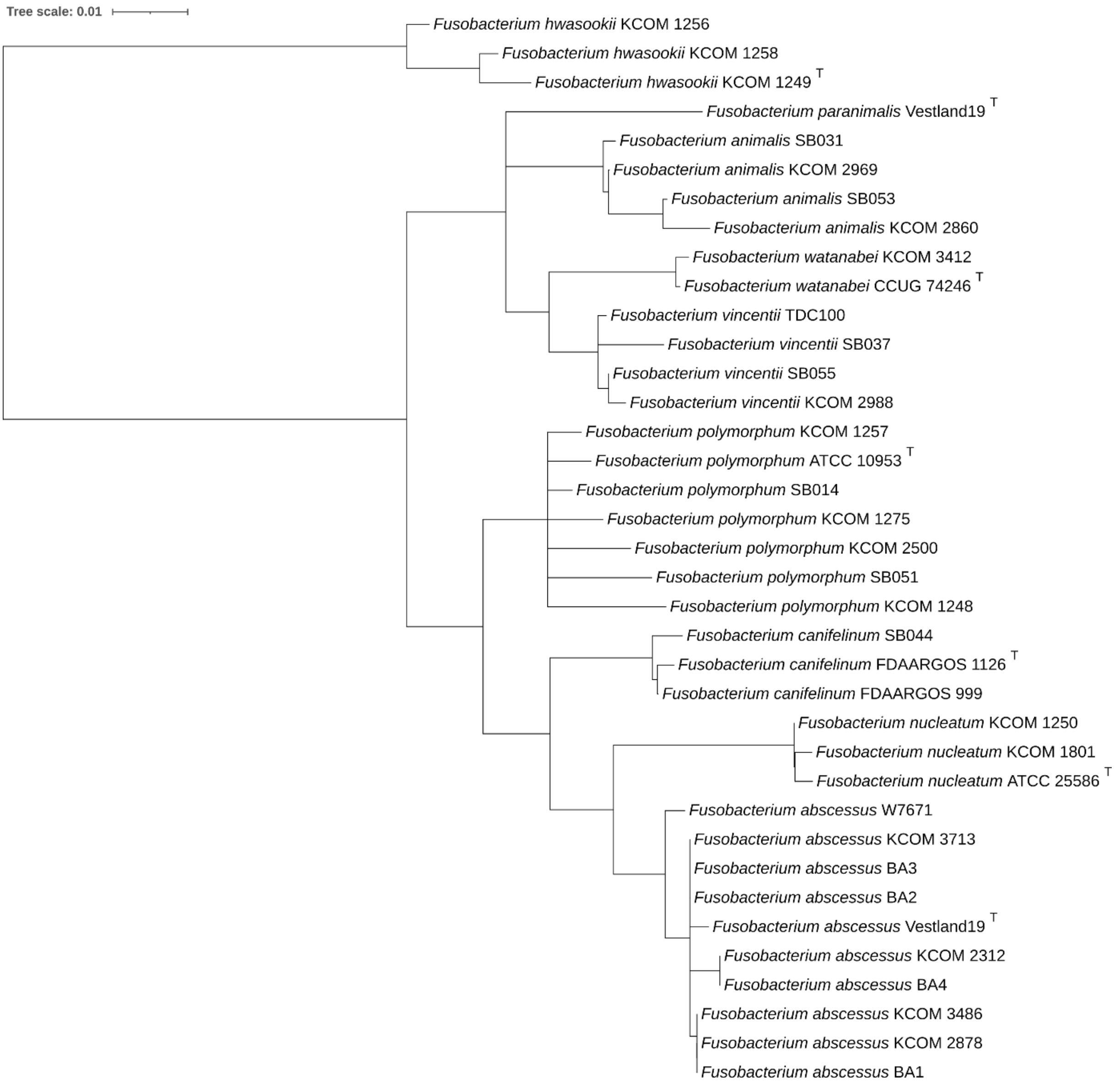

Previous *F. nucleatum* C2/*Fusobacterium* sp. HMT-203 are renamed as *F. abscessus*.

Sequences amplified directly from brain-abscess samples are named *F. abscessus* BA1-4.
